# Utility of parentage‐based tagging for monitoring Coho salmon (*Oncorhynchus kisutch*) in the interior Columbia River basin

**DOI:** 10.1111/eva.13607

**Published:** 2023-12-08

**Authors:** Rebekah L. Horn, Hayley M. Nuetzel, Becky Johnson, Cory Kamphaus, Jon Lovrak, Kraig Mott, Todd Newsome, Shawn R. Narum

**Affiliations:** ^1^ Columbia River Inter‐Tribal Fish Commission, Hagerman Genetics Lab Hagerman Idaho USA; ^2^ Columbia River Inter‐Tribal Fish Commission Portland Oregon USA; ^3^ Nez Perce Tribal Fisheries Grangeville Idaho USA; ^4^ Yakama Nation Fisheries Toppenish Washington USA; ^5^ Conferdated Tribes of the Umatilla Indian Reservation Pendleton Oregon USA

**Keywords:** Coho salmon, effective population size, genetic diversity, genetic structure, hatchery propagation, PBT

## Abstract

By the 1980s, after decades of declining numbers in the mid‐1900s, Coho salmon (*Oncorhynchus kisutch*) were considered extirpated from the interior Columbia River. In the mid‐1990s, the Confederated Tribes of the Umatilla Indian Reservation, the Confederated Tribes and Bands of the Yakama Nation, and the Nez Perce Tribe began successful reintroduction programs of Coho salmon upstream of Bonneville Dam, but which were initially sourced from lower Columbia River hatcheries. Here we present the first Coho salmon parentage‐based tagging (PBT) baseline from seven hatchery programs located in the interior Columbia River basin, and two sites at or downstream of Bonneville Dam, composed of over 32,000 broodstock samples. Analyses of baseline collections revealed that genetic structure followed a temporal pattern based on 3‐year broodlines rather than geographic location or stocking history. Across hatchery programs, similar levels of genetic diversity was present. The PBT baseline provided multiple direct applications such as identification of origin for Coho salmon collected in a mixed stock at Priest Rapids Dam and the detection of the proportion and distribution of hatchery‐origin fish on the spawning grounds in the Methow River basin. The PBT baseline for Coho salmon is freely available for use and can be downloaded from FishGen.net.

## 1 INTRODUCTION

Over the past century, salmon populations in the Columbia River Basin (CRB) have experienced drastic declines from historical abundances due to habitat degradation, over‐harvest and the construction of hydropower dams (Kareiva et al., 2000; Paquet et al., 2011; Williams, 2008). These interacting stressors have led to extirpations across species and regions, with an estimated 117 of 333 historical populations within the CRB considered extirpated (CBP, 2020). Coho salmon (*Oncorhynchus kisutch*) throughout the interior CRB region were deemed functionally extinct by the 1980s following failed attempts to rebuild Coho salmon populations in the mid‐1900s via stocking and transfer of lower Columbia River (LCR) eggs to hatchery facilities in the mid‐Columbia Region (Galbreath et al., 2014; Nehlsen et al., 1991). However, the Treaty Tribes—the Nez Perce Tribe (NPT), the Confederated Bands and Tribes of the Umatilla Indian Reservation (CTUIR), the Confederated Tribes of the Warm Springs Reservation of Oregon (CTWSRO) and the Confederated Tribes and Bands of the Yakama Indian Reservation (YN)—quickly responded to the loss of this critical cultural resource and exercised their Treaty rights to cooperatively develop the Columbia River Fish Management Plan in 1988. This plan affirmed their co‐management and harvest allocation rights alongside state and federal parties, while also providing provisions to utilize artificial propagation to rebuild upper river salmon runs (CRFMP, 1987). While the resulting Coho salmon reintroduction efforts in the interior CRB region had to again rely on LCR stocks to initiate artificial propagation, they differed from earlier reintroduction attempts in two notable ways: these efforts were spearheaded by several of the Treaty tribes and all program managers began their respective reintroductions with the intention of developing localized stocks that would not require continual supplementation with LCR stock fish.

The first tribally managed Coho salmon reintroduction programs began In the mid‐1990s, with the YN managing reintroduction to the Wenatchee, Methow and Yakima rivers, the CTUIR managing reintroduction to the Umatilla River and the NPT managing reintroduction to the Clearwater River (Galbreath et al., 2014). To achieve their long‐term programmatic goal of developing a localized stock, each hatchery program sought to eventually only source broodstock from in‐basin, returning adults. This strategy promotes local adaptation because individuals within the basin have encountered and survived the selective pressures unique to the reintroduced habitat, making them potentially more suitable for spawning within that system compared to out‐of‐basin fish (Brannon et al., 2004). In fact, in less than four generations, the reintroduction programs for Coho salmon in the Methow, Wenatchee, Umatilla and Clearwater rivers were able to stop sourcing broodstock from lower Columbia River hatcheries, as sufficient numbers of adults returned volitionally to interior Columbia River sites (Campbell et al., 2017; Galbreath et al., 2014).

Moreover, several of these programs are motivated by conservation as well as harvest augmentation goals, and as such, implement additional best practices for population recovery and sustainability (Flagg & Nash, 1999; Hatchery Scientific Review Group (HSRG), 2009; Naish et al., 2007). This includes the incorporation of natural‐origin (NOR) fish into broodstock, where possible, following the integrated broodstock model, which maintains gene flow between the hatchery‐reared and naturally spawning population components (Flagg, 2015; Flagg & Nash, 1999; Paquet et al., 2011). Managing hatchery broodstock composition in this manner requires returning fish be marked so that NOR and hatchery‐origin (HOR) fish are distinguishable. This has historically been achieved by external markings such as an adipose clip or tagging via a coded wire tag (CWT) (Johnson, 2004; Paquet et al., 2011). Releases of Coho salmon in the CRB from 2012 have variable tagging proportions, from 0% to 23% released with CWT and 77%–100% released with an adipose clip. Parentage‐based tagging (PBT) is a comparatively newer method of identifying origin and utilizes parent‐offspring relationships to infer both an individual's origin and age at return (Anderson & Garza, 2006; Steele et al., 2019). This approach requires first that hatchery broodstock be tissue sampled for genotyping of parents, and then offspring can be sampled and genotyped at any life stage (juvenile to adult). Parent‐offspring relationships are inferred using pedigree reconstruction tools, which then informs the offspring's origin and age. A PBT based management system generates very similar information and can be less expensive than deploying CWTs (Beacham, Wallace, Jonsen, McIntosh, Candy, Willis, Lynch, Moore, et al., 2019). In addition, PBT typically has higher tagging rates compared to CWTs and can accurately identify stray fish among hatcheries and the composition of mixed stocks in fishery captures (Beacham et al., 2020; Beacham, Wallace, Jonsen, McIntosh, Candy, Willis, Lynch, Moore, et al., 2019; Beacham, Wallace, Jonsen, McIntosh, Candy, Willis, Lynch, & Withler, 2019; Horn et al., 2023; Steele et al., 2019).

Hatchery stocks of Coho salmon in the CRB have routinely utilized adipose clips and CWTs, but widespread adoption of PBT methods has been limited. However, several of the Coho salmon reintroduction programs initiated by the tribes in the interior CRB have been collecting and using PBT data since 2012. While the inference provided by PBT expands as more Coho salmon hatcheries opt into the PBT method for program monitoring, this enables several questions of interest to be addressed among participating hatcheries. Here we present the first PBT baseline for interior Columbia River Coho salmon and detail the nine hatchery programs contributing samples to the baseline. Using the information contained within the baseline, we address the following objectives: (1) quantify the genetic diversity within and among hatchery programs and spawn years, (2) characterize the genetic differentiation between hatchery programs, (3) estimate the effective number of breeders each spawn year and (4) determine the broodstock composition by origin of each hatchery. We also demonstrate the utility of this PBT approach for specific applications in the interior CRB including estimates of hatchery‐origin fish present on the spawning ground in the Methow River and stock identification from a mixed collection at Priest Rapids Dam. Lastly, we describe potential future applications of the Coho salmon PBT baseline.

## 2 MATERIALS AND METHODS

### 2.1 Hatchery programs

#### 2.1.1 Umatilla River

Coho salmon re‐introductions in the Umatilla River basin are overseen by the CTUIR and the Oregon Department of Fish and Wildlife (ODFW). Until 2009, Coho salmon reared for release in the Umatilla River were initially collected from Bonneville Hatchery, but starting in 2010, broodstock was composed of adults that returned to the Three Mile Falls Dam Facility (TMD) on the Umatilla River (Oregon Department of Fish and Wildlife/Confederated Tribes of the Umatilla Indian Reservation, 2010). After spawning at TMD, fish were reared at Cascade Hatchery and acclimated at the Pendleton Acclimation facility on the Umatilla River.

#### 2.1.2 Mid‐Columbia River

The YN's goals in the Mid‐Columbia River are to restore naturally spawning Coho salmon populations and enable fisheries for this species. The Wenatchee River program was sourced from several LCR stocks starting in 1999 and within 1–2 generations, fish were returning volitionally in sufficient numbers to permit collection of local brood (Campbell et al., 2017; Yakama Nation Fisheries Resource Management, 2017). Collection of broodstock for the Wenatchee River program primarily occurs at Tumwater and Dryden dams, with fish returning to Leavenworth National Fish Hatchery (NFH) as a backup collection as needed. Spawning for the Wenatchee River stock take place at Leavenworth NFH. Rearing of Coho salmon to pre‐smolt size takes place at Cascade FH and Willard NFH until smolts are transported and acclimated in several ponds in the upper watershed (Yakama Nation Fisheries Resource Management, 2017).

The Methow River stock originated from sources in the LCR starting in 1997 and like the Wenatchee River program, had volitionally returning adults within a few generations (Campbell et al., 2017; Yakama Nation Fisheries Resource Management, 2017). Collection of broodstock for the Methow River program occurs solely at Wells Dam. Past collection sites also included Wells Fish Hatchery (FH), Methow FH and Winthrop NFH. Spawning for the Methow River stock takes place at Winthrop NFH. Rearing takes place at Cascade FH, Willard NFH and Winthrop NFH and smolts are acclimated in several semi‐natural and constructed ponds in the Methow River basin major tributaries (Twisp, Chewuch and upper Methow River basins) (Yakama Nation Fisheries Resource Management, 2017).

The Little White Salmon NFH/Willard NFH complex on the Little White Salmon River rear Coho salmon for transfers to the YN program in the mid‐Columbia River basin if broodstock numbers fall short. No native stock of Coho salmon remains in the Little White Salmon River as backwater from Bonneville Dam (1938) covered all areas that were suitable for Coho salmon spawning (US Fish and Wildlife Service, 1999). Therefore, broodstock sources were obtained from Lower Kalama FH, Cascade FH, Bonneville FH, Speelyai FH and/or Eagle Creek NFH. The Little White Salmon/Willard NFH Coho salmon from 2015 and 2016 included in the PBT baseline are ones that were outsourced from Little White Salmon/Willard NFH for the YN program due to low returns across the entire CRB in those years.

#### 2.1.3 Yakima River

The YN have also reintroduced Coho salmon to the Yakima River from LCR sources starting in the mid‐1990s (Bosch et al., 2007). In‐basin broodstock collection takes place at Prosser Dam and Roza Dam located on the Yakima River. Spawning, incubation and rearing takes place at Prosser FH (Yakama Nation, 2010) and at the Melvin R. Sampson (MRS) Coho Facility in Ellensburg, WA. The broodstock program at Prosser FH operates as a segregated program and seeks to provide fish for tribal harvest opportunities. Previously, the Prosser facility released 50% YN‐origin Coho salmon and 50% Eagle Creek NFH stock but has begun to phase out use of outside broodstock starting in brood year 2023. Additionally, the YN program is integrating a small late‐run Coho salmon stock into the Prosser FH stock with the first late‐run brood taken from Ringold Springs Hatchery in 2022.

The YN have recently initiated another Coho hatchery program in the Yakima River basin, which operates as an integrated broodstock program and seeks to contribute to harvest as well as population recovery in the upper Yakima River. The program has outlined broodstock management goals in distinct phases and is currently in Phase 3, which seeks to use only in‐basin fish and incorporate 30% NOR fish into broodstock. Broodstock for this program are collected at Prosser and Roza dams and then spawned at the MRS Coho Facility. The first year of spawning occurred in 2020, with all broodstock being sourced from Prosser Dam (i.e., naturalized Yakima River Coho salmon). In spawn year 2021, managers were able to incorporate natural‐origin (NOR) fish returning to Roza into spawning matrices, alongside fish collected at Prosser Dam. Juveniles are released as both parr and smolts in various tributaries and mainstem reaches of the upper Yakima and Naches rivers.

#### 2.1.4 Clearwater River

Coho salmon were extirpated from the Clearwater River basin in Idaho in 1927 following the construction of the Lewiston Dam, but the NPT began reintroducing Coho salmon to the Clearwater River in 1994. From 1994 to 1998, broodstock was sourced from lower Columbia River hatcheries including Cascade FH, Bonneville FH, Willard NFH and Eagle Creek NFH. By 1999, managers were able to incorporate adults returning to the Clearwater River in the broodstock, but still relied on LCR stocks, particularly Eagle Creek NFH, to supplement and achieve sufficient spawning numbers. The first year in which broodstock was comprised entirely of in‐basin returns was 2009, and this has been the protocol every year since, except in years when returns were low. Broodstock are collected at Kooskia NFH and from a weir on Lapwai Creek. In low return years, broodstock can also be collected at Lower Granite Dam and Dworshak NFH. All spawning takes place at Dworshak NFH, and rearing occurs at both Dworshak NFH and Eagle Creek NFH. Smolts are acclimated at Kooskia NFH or Lapwai Creek before being released into Clear Creek or Lapwai Creek.

#### 2.1.5 Columbia River

Bonneville FH serves to mitigate for the loss of Coho salmon by contributing to sport, tribal and commercial fishing opportunities in the Columbia River basin (Oregon Department of Fish and Wildlife, 2016). Bonneville FH spawn Tanner Creek Coho salmon, which are classified as hatchery stock 14 in the Lower Columbia River Coho Evolutionary Significant Unit (ESU). Bonneville FH operates as a segregated facility and only accepts adipose‐clipped fish as broodstock. Returning adults are captured for broodstock from those that volitionally ascend the fish ladder at Bonneville FH. After the spawning of adults at Bonneville FH, fertilized green eggs are sent to Cascade FH for incubation and early rearing. Final rearing takes place at Bonneville FH and fish are released directly into Tanner Creek. Cascade FH also assists with the early rearing (eyed egg to pre‐smolt stage) of fish from the Wenatchee River basin, Methow River basin, the Umatilla River and the NPT's Lostine River re‐introduction program (Oregon Department of Fish and Wildlife, 2020).

#### 2.1.6 Clackamas River

Eagle Creek NFH is located on Eagle Creek, a tributary to the Clackamas River in Oregon, and serves to mitigate for the loss of salmon due to the construction of Bonneville Dam and habitat loss and to supplement Coho salmon harvest in the Lower Columbia River. Fish volitionally return to the fish ladder located at Eagle Creek NFH and are spawned onsite. To date, PBT samples have only been collected and genotyped from Eagle Creek NFH from spawn year 2019. The samples have been included as part of this PBT dataset. This hatchery rears Coho salmon for release into Eagle Creek, but also to assist with the re‐introductions into the Wenatchee, Methow, Yakima, Umatilla, and Clearwater rivers by the Columbia River treaty tribes (Silver et al., 2020). All transferred fish are adipose clipped and/or tagged with a CWT before transfer and a small proportion of the fish transferred to the Clearwater River are PIT tagged (1%–2%). In addition to Eagle Creek NFH, Bonneville FH and Cascade FH that are located below Bonneville Dam, Kalama FH and Washougal FH also spawn and rear Coho salmon for releases above Bonneville Dam to the interior CRB, mostly to the Klickitat River, but are not part of the PBT baseline.

#### 2.1.7 Genetic data

**FIGURE 1:**
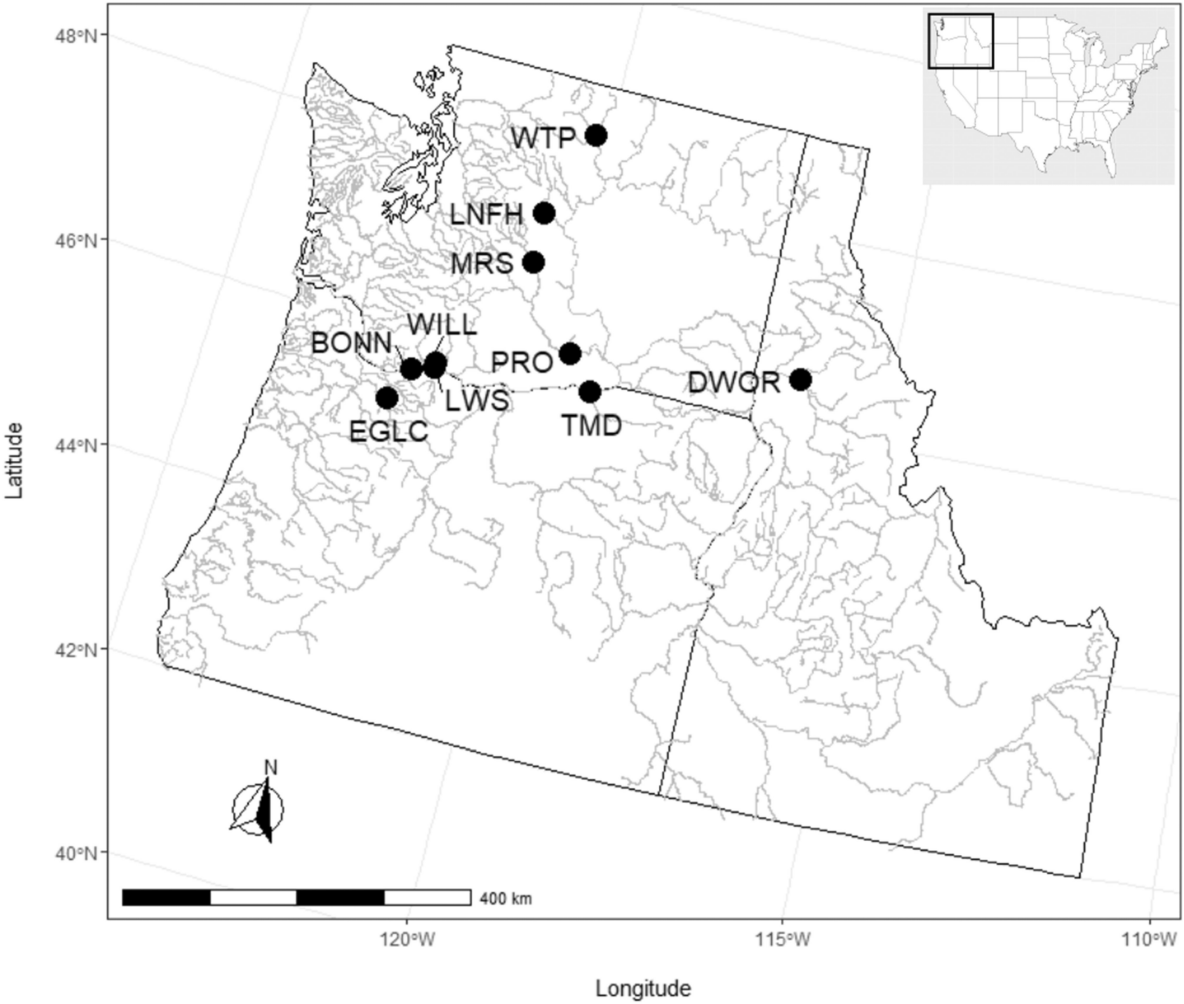
Map showing the location of Coho salmon hatcheries for which PBT samples have been collected and included in the baseline. This map also includes the location of the Melvin R. Sampson Coho Facility (MRS) operated by the Yakama Nation that began PBT broodstock sampling in 2021, but which has not yet been incorporated into the PBT baseline. For a list of hatchery abbreviations, see Table 1.

The Coho salmon PBT baseline consists of samples from seven hatcheries from the interior Columbia River (above Bonneville Dam) and two hatcheries located at or below Bonneville Dam (Figure 1). Samples for PBT have been collected, genotyped and included in the PBT baseline from Leavenworth NFH and Winthrop NFH from 2012 to 2020, TMD and Dworshak NFH from 2015 to 2020, Prosser FH from 2016 to 2020, Bonneville FH and Eagle Creek NFH from 2019, Little White Salmon NFH from 2015 and 2016 and Willard NFH from 2016 (Table 1). During broodstock spawning at the hatcheries, a hole punch or scissors are used to collect a piece of fin tissue from each male and female spawned. Fin tissues are placed onto Whatman chromatography sheets for storage and shipment. Genomic DNA was extracted from all samples using a Chelex protocol (Sigma‐Aldrich) and broodstock samples were genotyped using the GT‐seq method described in Campbell et al. (2015).

**TABLE 1:** A list of hatcheries at which Coho salmon are spawned and that have sampled broodstock for parentage‐based tagging (PBT). Samples refers to the number of samples received for genotyping.

Hatchery	Abbrev	Operator	SY	Samples	Failed	Duplicate	Tag rate
Bonneville FH	BONN	ODFW	2019	1183	15	6	0.965
Eagle Creek NFH	EGCL	USFWS	2019	1757	17	8	0.971
Leavenworth NFH	LNFH	YN/USFWS	2012	873	60	1	0.865
Leavenworth NFH	LNFH	YN/USFWS	2013	867	19	2	0.952
Leavenworth NFH	LNFH	YN/USFWS	2014	795	274	6	0.420
Leavenworth NFH	LNFH	YN/USFWS	2015	854	64	1	0.854
Leavenworth NFH	LNFH	YN/USFWS	2016	1025	29	2	0.940
Leavenworth NFH	LNFH	YN/USFWS	2017	1034	15	3	0.965
Leavenworth NFH	LNFH	YN/USFWS	2018	1118	3	4	0.988
Leavenworth NFH	LNFH	YN/USFWS	2019	1188	7	1	0.987
Leavenworth NFH	LNFH	YN/USFWS	2020	982	29	4	0.934
Little White Salmon NFH	LWS	USFWS	2015	6	0	0	1.000
Little White Salmon NFH	LWS	USFWS	2016	84	5	0	0.885
Dworshak NFH	DWOR	NPT	2015	1030	32	3	0.933
Dworshak NFH	DWOR	NPT	2016	1353	59	16	0.892
Dworshak NFH	DWOR	NPT	2017	1372	21	6	0.961
Dworshak NFH	DWOR	NPT	2018	598	9	1	0.967
Dworshak NFH	DWOR	NPT	2019	1370	49	8	0.919
Dworshak NFH	DWOR	NPT	2020	1079	1	2	0.994
Prosser FH	PRO	YN	2016	488	44	16	0.769
Prosser FH	PRO	YN	2017	754	228	18	0.454
Prosser FH	PRO	YN	2018	579	54	3	0.813
Prosser FH	PRO	YN	2019	1001	74	12	0.836
Prosser FH	PRO	YN	2020	1184	87	8	0.846
Three Mile Falls Dam	TMD	CTUIR/ODFW	2015	894	5	0	0.989
Three Mile Falls Dam	TMD	CTUIR/ODFW	2016	513	11	0	0.958
Three Mile Falls Dam	TMD	CTUIR/ODFW	2017	532	7	0	0.974
Three Mile Falls Dam	TMD	CTUIR/ODFW	2018	591	2	1	0.990
Three Mile Falls Dam	TMD	CTUIR/ODFW	2019	444	6	1	0.969
Three Mile Falls Dam	TMD	CTUIR/ODFW	2020	482	2	0	0.992
Willard NFH	WILL	USFWS	2016	36	1	0	0.945
Winthrop NFH	WTP	YN/USFWS	2012	638	116	1	0.667
Winthrop NFH	WTP	YN/USFWS	2013	241	9	2	0.911
Winthrop NFH	WTP	YN/USFWS	2014	433	46	1	0.795
Winthrop NFH	WTP	YN/USFWS	2015	530	8	3	0.959
Winthrop NFH	WTP	YN/USFWS	2016	198	4	0	0.960
Winthrop NFH	WTP	YN/USFWS	2017	1149	44	0	0.925
Winthrop NFH	WTP	YN/USFWS	2018	1009	13	1	0.972
Winthrop NFH	WTP	YN/USFWS	2019	1165	7	0	0.988
Winthrop NFH	WTP	YN/USFWS	2020	748	9	0	0.976

*Note*: Each hatchery facility is organized by spawn year (SY). The abbreviation (Abbrev) is the shortened name used in the figures; Operator indicates which agency operates the hatchery (CTUIR, Confederated Tribes of the Umatilla Indian Reservation; NPT, Nez Perce Tribe; ODFW, Oregon Department of Fish and Wildlife; USFWS, United States Fish and Wildlife Service; YN, Yakama Nation Tribes); Failed indicates the number of samples that failed genotyping at 90% or more of the SNP loci; Duplicate is the number of samples found to be a genetic duplicate to another sample; Tag Rate is the proportion of successfully genotyped samples per SY.

The SNP markers used for the Coho salmon PBT panel were developed from two primary sources. First previous Taqman assays were converted directly to amplicons with the same primers and were based on an ascertainment panel of 32 individuals covering much of the species' range (see Campbell & Narum, 2011 for details). Second, SNPs were selected from RAD loci in Coho salmon from an ascertainment panel that included lower Columbia stocks and those introduced in the mid‐Columbia River (Campbell et al., 2017). Given the general lack of population structure for Coho salmon in the Columbia River, markers were selected to represent random SNPs across the genome and relatively high heterozygosity that would enable PBT applications rather than other criteria such as *F*_ST_. Additional SNPs were added to the panel based on amplicons from Beacham et al. (2017). Initial testing involved 352 SNP markers from these combined sources and the current panel for Coho salmon PBT contains 235 SNP markers (Hess et al., 2021), with two being sex markers as previously described (Horn et al., 2020) (Table S1). During testing, markers were screened to avoid SNPs that demonstrated patterns of unresolvable duplicated loci, which were evident when more than three clusters of genotypes were observed at a locus. However, diverged duplicates were retained when the expected three genotypic classes could be scored with a correction factor that accounted for shifts in read numbers. Further guidance for scoring duplicated loci from sequencing is provided by McKinney et al. (2017). All samples and their genotypes have been deposited into FishGen.net (McCane et al., 2018), which is publicly accessible.

Individual genotypes were then filtered to remove samples with high incidences of missing data (10% missing genotypes), or which were found to be a duplicate of another sample in the broodstock. Two samples were designated as duplicates if they had 95% or more identical genotypes across all non‐missing markers. Failed and duplicated samples were assessed with the R package EFGLmh (https://github.com/delomast/EFGLmh). The tag rate for each hatchery and spawn year was then calculated using Method 3 of Satterthwaite et al. (2015).

Filtered genotype data was supplied to a pedigree reconstruction program (SNPPIT; Anderson, 2012) to determine broodstock composition (e.g., origin and total age, defined as the age from egg fertilization to spawning adult) of each hatchery by spawn year. Coho salmon from hatchery programs in the lower Columbia River generally return as 3‐year‐old fish but with a small portion of ‘jack’ males returning after 2 years (Smith et al., 2015). We used this life history to structure each hatchery's pedigree reconstruction analysis and identify the subset of years for which we had sufficient data to identify parent‐offspring relationships. For Leavenworth NFH and Winthrop NFH, this included spawn years 2015–2020 and 2018–2020 for Prosser FH, TMD and Dworshak NFH. Parent pair‐offspring trio assignments were assessed with the program SNPPIT (Anderson, 2012). A trio assignment was accepted if the false discovery rate was less than 0.1 and the LOD score was greater than 14. These parameters were chosen based on the previous testing and work in Chinook salmon (*O. tshawytscha*) and steelhead trout (*O. mykiss*) to minimize false positive assignments (Hargrove et al., 2021; Hess et al., 2016; Horn et al., 2023). Parentage assignment was not performed for Willard NFH and Little White Salmon NFH as data from the potential parent years, 2012 and 2013 were not included in the PBT baseline. Parentage assignment was performed for adults returning to Eagle Creek NFH and Bonneville FH in 2019 despite not having the parental year (2016 and 2017) represented in the baseline because these are downriver sites to which interior Columbia River Coho salmon may have strayed.

Population genetic metrics were assessed for each hatchery and spawn year using R v 3.5.2 and packages GPoppin (function ‘popgen’; https://github.com/StevenMicheletti/GPoppin) and diveRsity (function ‘divBasic’ for FIS values and confidence intervals; https://github.com/kkeenan02/diveRsity‐online). Since there were only six samples in the Little White Salmon NFH broodstock from 2015, those samples were omitted from population genetic analysis. Measures of heterozygosity [observed (*H*_*O*_) and expected (*H*_*E*_) heterozygosity] and the degree of relatedness (*F*_IS_) were assessed for all hatcheries and spawn years. Genetic differentiation (*F*_ST_) was calculated among hatcheries and spawn years and a principal components analysis (PCA) was used to assess the level of population genetic structure. The effective number of breeders for each spawn year (*N*_*b*_) was estimated with the linkage disequilibrium (LD) method (Jones et al., 2016; Waples, 2006) in the program NeEstimator V2.1 (Do et al., 2014).

#### 2.1.8 Applications of PBT—Priest Rapids dam

The YN mid‐Columbia River program began PIT tagging and taking genetic samples for PBT of Coho salmon adults returning over Priest Rapids Dam on the Columbia River starting in 2019. The intent was to develop better estimates of adult escapement to the Upper Columbia River. Coho salmon redds are difficult to locate and using redd counts alone can underestimate escapement. The combination of PIT tag data and PBT sampling has the potential to increase the accuracy of adult escapement to the mid‐ to upper‐reaches of the Columbia River. In 2019 and 2020, 1638 and 2218 Coho salmon adults were sampled as they passed Priest Rapids Dam. Genotyping followed the methods outlined above for the panel of 235 SNPs. Samples that failed genotyping at a 90% threshold and duplicated samples were assessed with the R package EFGLmh (https://github.com/delomast/EFGLmh). Parentage analysis was performed with the program SNPPIT (Anderson, 2012). Sex assignment for each sample was facilitated by the sex markers present in the SNP panel (Horn et al., 2020).

#### 2.1.9 Applications of PBT—Methow River basin carcass sampling

Spawning ground surveys have been conducted in the Methow River basin since 2001 by the YN to estimate the proportion of hatchery and natural‐origin fish on the spawning grounds. Locations within the Methow River basin include the Methow River, Chewuch River, Twisp River, Wolf Creek, Hancock Springs Creek, Beaver Creek, Libby Creek, Gold Creek, Winthrop NFH back channel (Spring Creek), Methow Fish Hatchery outfall, 1890s Side Channel, Fender Mill Creek, Suspension and Little Suspension Creek. Out‐of‐basin tributaries include Chelan River, Beebe Springs, Foster Creek and tributaries to the Okanogan River. All carcasses are bio‐sampled to obtain PBT fin clips specific to each carcass. Carcasses are rated from one to three with one representing carcasses that are 0‐ to 1‐day‐old, two representing carcasses that are 2–4 days old, and three for those carcasses that are 5+ days old. Scales are taken to age the carcass and the snout is removed to check for and read a coded wire tag (CWT). Fork length and postorbital‐hypural lengths are also measured and recorded for each carcass. The genetic samples are prepped for GT‐seq following the same methods as outlined above and genotyped for the panel of 235 SNPs. Parentage analysis was conducted with SNPPIT (Anderson, 2012) after accounting for failed and duplicate samples with the R package EFGLmh (https://github.com/delomast/EFGLmh). A total of 164 samples were genotyped from the 2017 spawning ground survey, 83 from 2018, 81 from 2019 and 45 from 2020.

## 3 RESULTS

There are a total of 32,177 Coho salmon samples in the PBT baseline. Of those, 1485 failed genotyping thresholds (95.4% genotyping success rate) and 141 were found to be a duplicate of another sample. There were only three hatchery spawn years in which there was less than 85% genotyping success: Prosser FH from 2017 (228 samples failed genotyping out of 754), Leavenworth NFH from 2014 (274 samples failed genotyping out of 795), and Winthrop NFH from 2012 (116 samples failed genotyping out of 638) (Table 1). Data from all samples have been uploaded to FishGen.net and are the first Coho salmon PBT samples to be represented on this platform.

We identified parent assignments for 13,572 out of 20,737 samples (65%) from within the PBT baseline (Table S2). Assignment success ranged from 98% [spawn year (SY) 2018 TMD] to 0% (SY2019 Eagle Creek NFH). The 0% assignment success rate for SY2019 from Eagle Creek NFH was due to there being no representation of the predominant parental year (SY2016) in the baseline and also suggests no fish from upstream Coho salmon programs strayed to Eagle Creek NFH in 2019 and were incorporated as broodstock. Most returning adult fish that were collected for broodstock and spawned were 3‐year‐olds with only a small proportion of jacks being incorporated each spawn year (Table S2). For Prosser FH in SY2018, the finding of 100% age‐2 broodstock composition was an artefact of the sample collection, as the majority of the individuals returning in 2018 were likely descended from parents spawned at Prosser FH in 2015, which was not represented in the PBT baseline.

Parentage analyses revealed a few instances where broodstock was assigned to parents from outside hatchery programs with higher frequency than usual due to stock transfers in low return years. For Prosser FH SY2018 and SY2019, fish were predominately assigned to parents from the Little White Salmon/Willard NFH complex. The Coho salmon return in 2016 was low across the entire basin and many programs were short on broodstock goals. Coho salmon that returned to the Little White Salmon/Willard NFH in 2016 were opportunistically spawned and offspring were outsourced to the YN for eventual release into the Yakima River. At Dworshak NFH in 2018, 197 fish out of 588 fish (34%) were assigned to parents from TMD in SY2015. There was a low return year in 2015 for Coho salmon returning to the Clearwater River, therefore, eggs were acquired from the Umatilla River stock for rearing at Dworshak NFH and released into the Clearwater River. At Bonneville FH in 2019, 98 fish were assigned to the TMD program from SY2016, which accounted for approximately 8% of broodstock spawned at Bonneville FH. Besides these instances, the overall rate of outside brood was low among programs (Table S2) as each program has prioritized local broodstock collections when possible. However, incomplete sampling across hatcheries and spawn years for Coho salmon in the interior Columbia River, makes it difficult to fully account for stock transfers as well as the number of NOR fish incorporated as broodstock. For example, an unmarked/untagged fish without a parentage assignment may be a NOR fish, but it also may be descended from a hatchery program that does not participate in PBT sampling.

**FIGURE 2:**
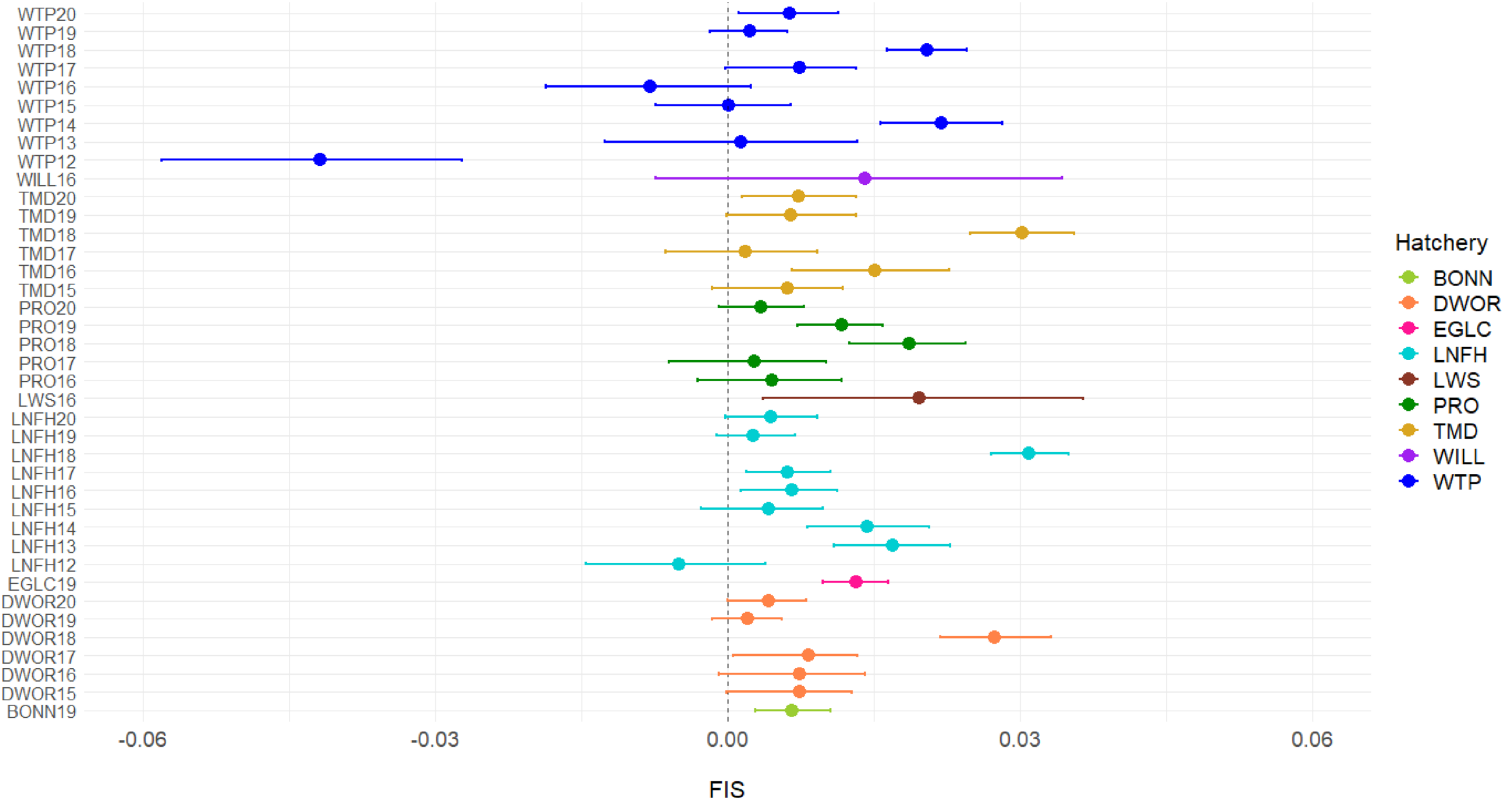
The *F*_IS_ values (circle) with their associated 95% confidence intervals for each Coho salmon hatchery and brood year. A list of hatchery abbreviations can be found in Table 1.

The observed and expected heterozygosity was consistent across hatcheries and spawn years (average *H*_*O*_ = 0.437, *H*_*E*_ = 0.438) (Figure S1). In almost all years and hatcheries, the *H*_*O*_ was smaller than the *H*_*E*_, indicating heterozygote deficit in most cases. This is also reflected in *F*_IS_ values significantly greater than zero for 18 of the 39 programs [46.2%; significance categorized by 95% confidence intervals (CI) nonoverlapping with zero], which can indicate a higher degree of mating among related individuals (Figure 2). There was one collection (WTP12) in which the *F*_IS_ value was significantly less than zero (Figure 2).

**FIGURE 3:**
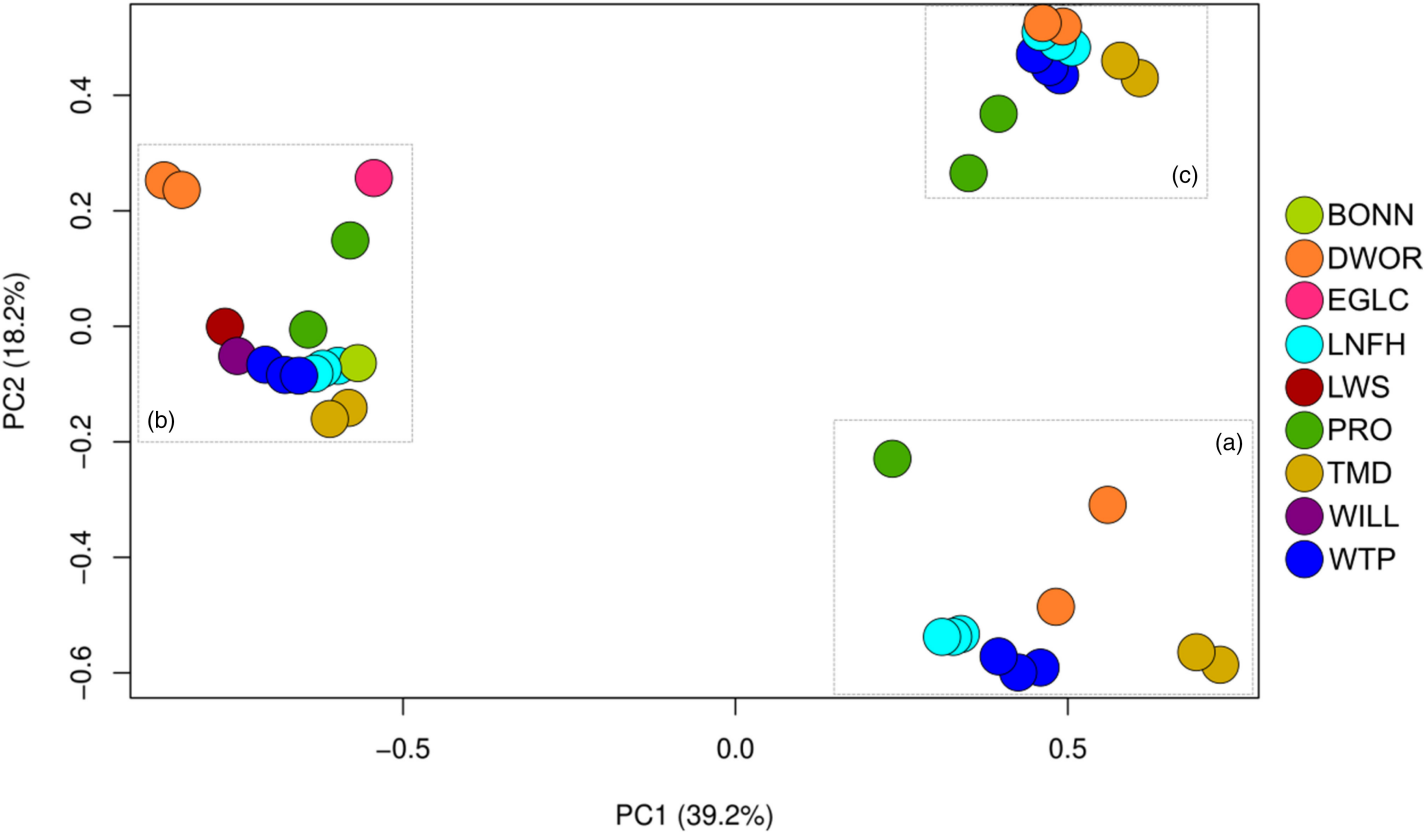
A principal components analysis (PCA) of all Coho salmon hatcheries and brood years. Dashed, grey boxes highlight the clustering of the ‘A’ broodline (spawn years 2012, 2015 and 2018), the ‘B’ broodline (2013, 2016 and 2019) and the ‘C’ broodline (spawn years 2014, 2017 and 2020). For a list of hatchery abbreviations, see Table 1.

**FIGURE 4:**
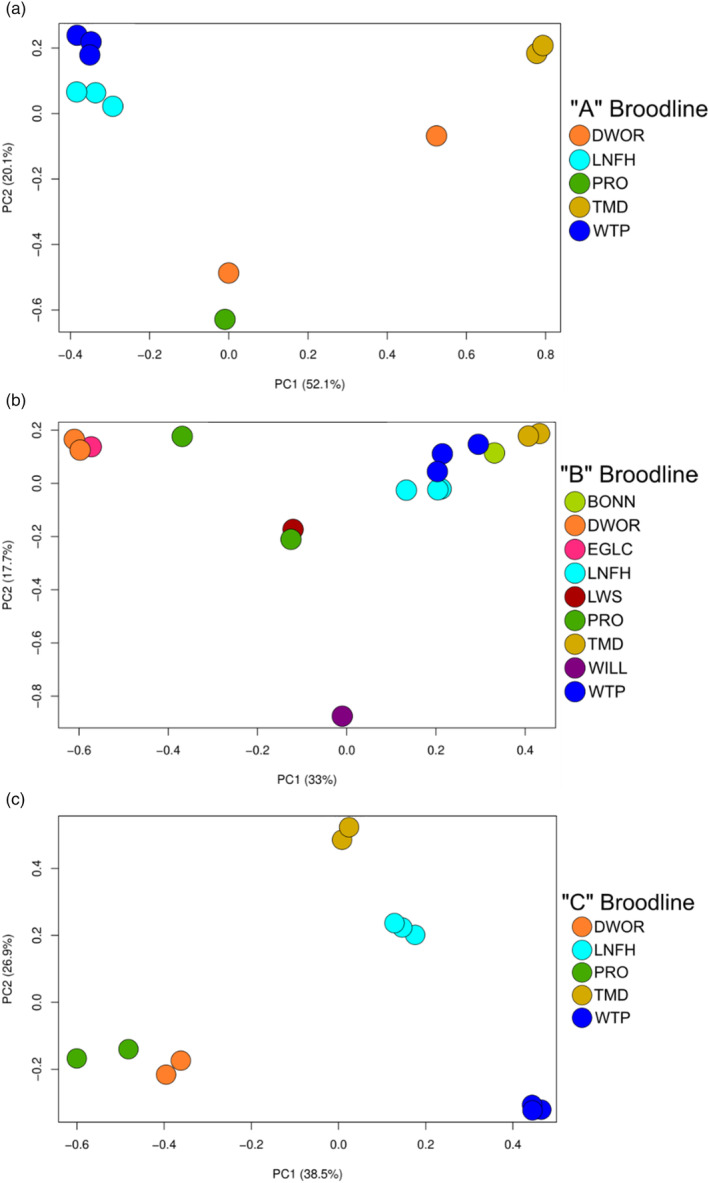
Principal components analysis of each Coho salmon hatchery and brood year within broodlines: (a) broodline ‘A’ (2012, 2015 and 2018); (b) broodline ‘B’ (2013, 2016 and 2019); (c) broodline ‘C’ (2014, 2017 and 2020). For a list of hatchery abbreviations, see Table 1.

The PCA (Figure 3) detected a stronger signal of genetic structuring by age class (broodlines) rather than among hatchery programs, which was also supported by the *F*_ST_ values (Figure S2). Samples from spawn years 2012, 2015 and 2018 clustered together (hereafter referred to as the ‘A’ broodline, following notation by Campbell et al., 2017), spawn years 2013, 2016 and 2019 clustered together (‘B’ broodline) and spawn years 2014, 2017 and 2020 formed a third cluster (‘C’ broodline). As in the Campbell et al. (2017) study, which only included fish from the Wenatchee River, the B broodline is more distinct from broodlines A and C (Figure 3) in all hatchery programs in this study. The PCA analysis was repeated for each separate broodline to observe possible population structure among hatcheries within broodlines. Within all broodlines, the Dworshak NFH and Prosser FH stocks were most similar and the Winthrop NFH and Leavenworth NFH stocks were more closely related in the A and B broodlines (Figure 4). Fish from the TMD program were generally more distinct from the others, except for the B broodline, where they grouped with Leavenworth NFH, Winthrop NFH and Bonneville NFH stocks (Figure 4b).

**FIGURE 5:**
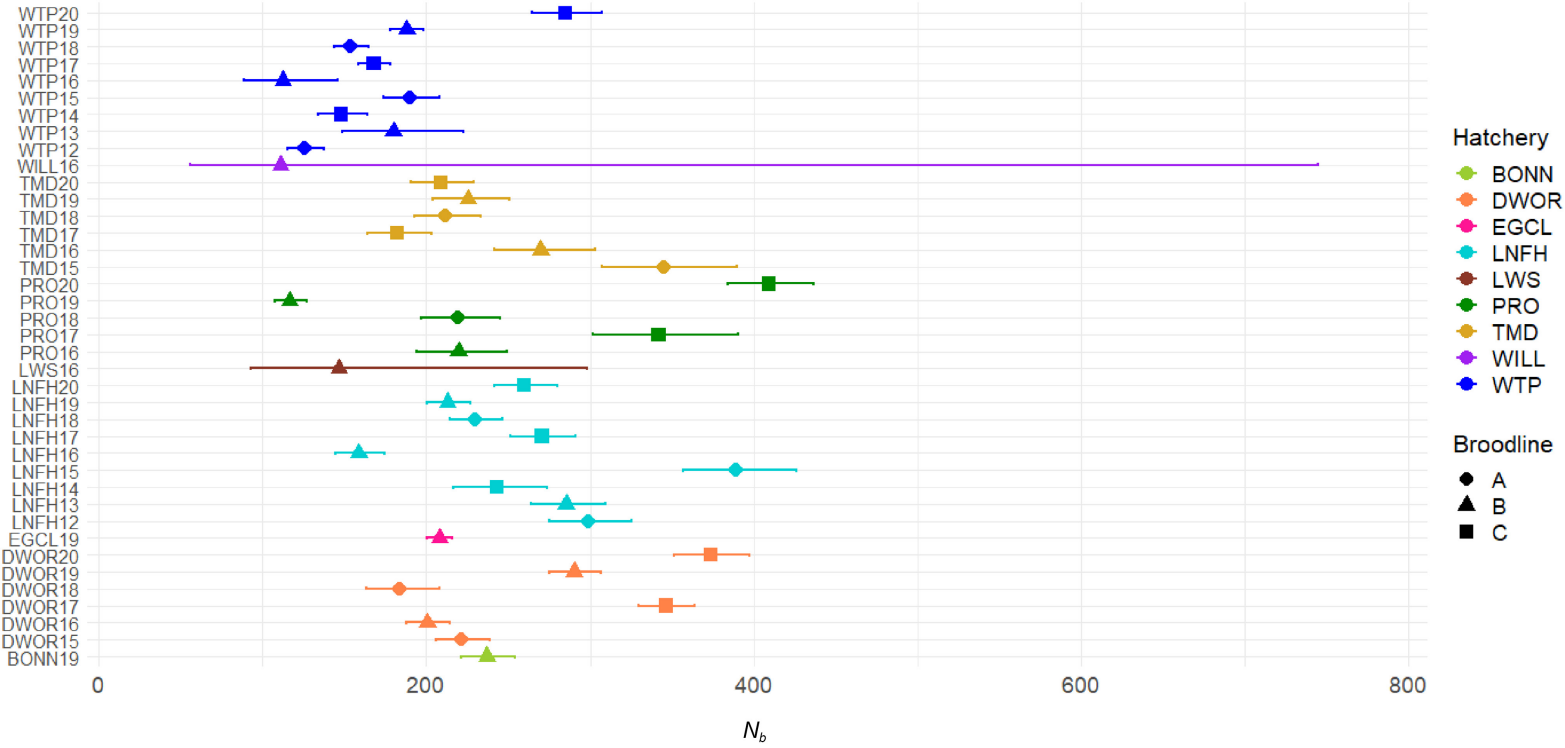
The effective number of breeders (*N*_*b*_) for each Coho salmon hatchery and brood year. The ‘A’ broodline contains spawn years 2012, 2015 and 2018, the ‘B’ broodline contains spawn years 2013, 2016, and 2019, and the ‘C’ broodline contains 2014, 2017 and 2020. For a list of hatchery abbreviations, see Table 1.

The effective number of breeders (*N*_*b*_) ranged from 110 (confidence interval, CI: 58–745) to 410 (CI: 384–437) per hatchery and spawn year (Figure 5). There were no overall trends for increasing or decreasing *N*_*b*_ within a hatchery over multiple spawn years, but it was notable that *N*_*b*_ was greater in the ‘C’ broodline from Prosser FH and Dworshak NFH compared to the ‘A’ or ‘B’ broodlines from the same hatcheries (Figure 5). There was a total of 6924 parents identified (3429 males; 3495 females) out of a potential of 18,321 parents (total from spawn years 2012–2018). Spawn year 2018 was included because of the possibility of sampling jacks in SY2020; however, most offspring from SY2018 crosses will return in 2021, which was not included in this analysis. For the males, 1152 produced one offspring that returned to a hatchery and was incorporated into the broodstock (33.0%), and for females, 1116 produced one offspring (32.5%) (Figure S3).

### 3.1 Priest Rapids dam

**FIGURE 6:**
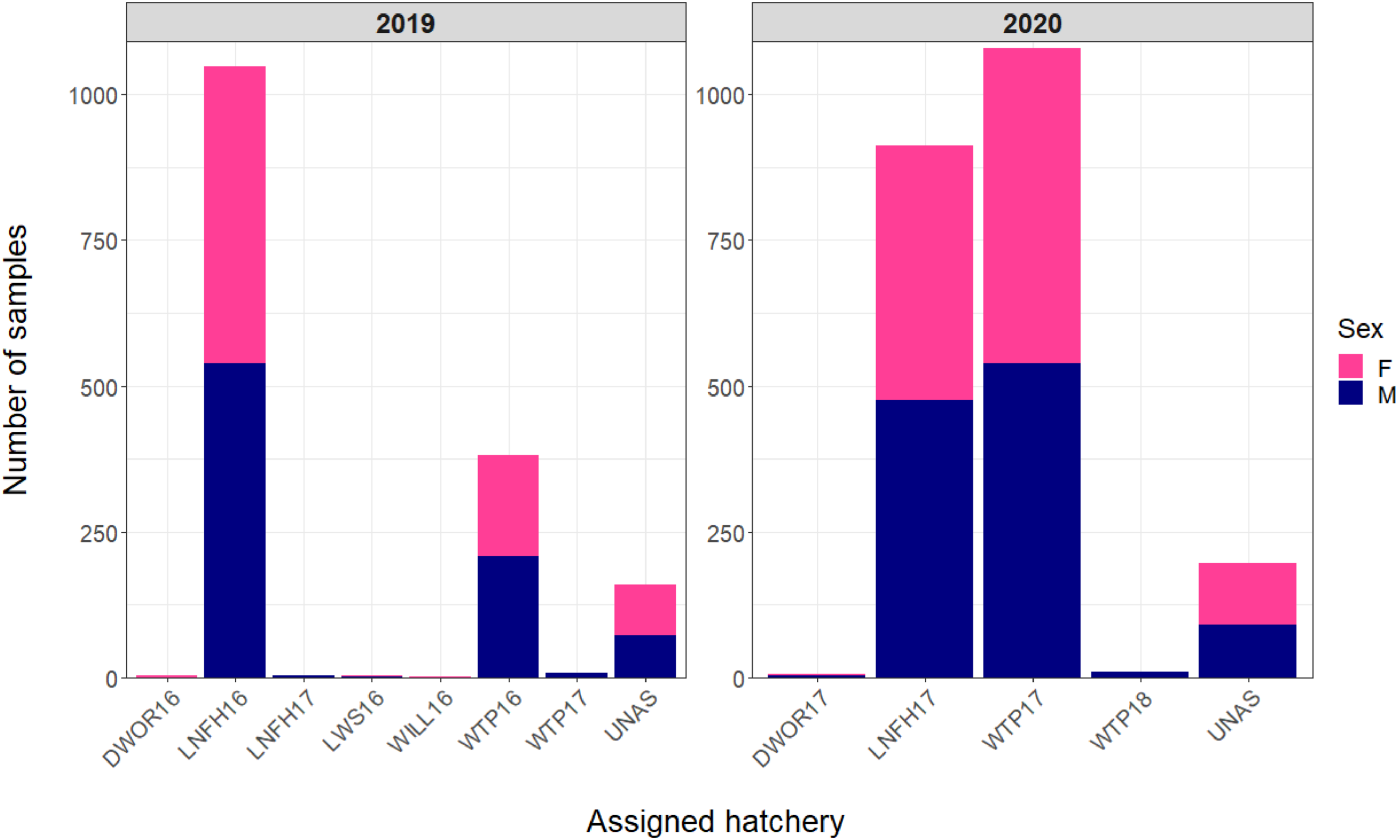
The number of returning adult Coho salmon sampled at Priest Rapids Dam in 2019 and 2020 that were assigned with PBT to a source hatchery (*x*‐axis). Male samples (M) are colour‐coded as blue and female samples (F) are colour‐coded as pink. The list of hatchery abbreviations is located in Table 1; UNAS, unassigned samples.

Of the 1638 samples genotyped from Priest Rapids Dam in 2019, there were 28 that failed genotyping and three samples that were a duplicate, leaving 1607 samples remaining for analysis. The majority of genotyped adults assigned to either Leavenworth NFH (*n* = 1047) or Winthrop NFH (*n* = 381) from SY2016, with more males (LNFH, *n* = 539; WTP, *n* = 508) returning compared to females (LNFH, *n* = 508; WTP, *n* = 172) (Figure 6). There were also a few male jack fish detected originating from Leavenworth NFH (*n* = 4) and Winthrop NFH (*n* = 7). There were five fish from the Little White Salmon/Willard NFH complex (*n* = 3 males, *n* = 2 females). Only three fish were identified as originating from a hatchery not located in the mid‐ to upper‐Columbia River (Dworshak NFH, all males) (Figure 6). There were 160 samples that did not assign with PBT, of which, 72 were males and 88 were females.

For the 2020 sample collection, there were nine samples that failed genotyping and three were found to be a duplicate, leaving 2206 samples for analysis. Most assignments were to Leavenworth NFH (*n* = 913) and Winthrop NFH (*n* = 1080) from SY2017 (Figure 6). There were more males that returned from Leavenworth NFH SY2017 (*n* = 476) compared to females (*n* = 437), but sex ratios were roughly equal for the returners originating from Winthrop NFH (*n* = 539 males, *n* = 541 females). There were 10 male jack fish that returned to Priest Rapids Dam in 2020 that originated from Winthrop NFH SY2018. Only six samples were found to have originated from an out‐of‐basin source (Dworshak NFH; *n* = 3 males, *n* = 3 females) (Figure 6). A total of 197 samples did not assign with PBT from 2020, 91 males and 106 females.

### 3.2 Methow River basin carcass sampling

Due to the degraded nature of carcass samples, there were many failed samples from each year: 84 of the 164 samples failed genotyping in 2017, 50 out of 83 failed in 2018, 30 of 81 failed in 2019 and 16 of the 45 failed genotyping in 2020. This resulted in 180 of the 373 total samples failing producing an overall genotyping success of 51.7%. The parentage results from the 2017 samples resulted in 60 PBT assignments and 20 unassigned samples. Of the 60 PBT assigned samples, 59 were to Winthrop NFH from SY2014 and one was to Leavenworth NFH from SY2013 indicating that it was a 4‐year‐old fish (Table 2). In 2018, there were 31 PBT assignments all to Winthrop NFH from SY2015 and two samples that did not assign with PBT (Table 2). In 2019, out of the 51 samples used in the parentage analysis, 23 of the PBT assignments were to Leavenworth NFH (SY2016), 24 were to Winthrop NFH (SY2016) and 4 were unassigned (Table 2). Lastly, in 2020, all PBT assignments were to Winthrop NFH from either SY2017 (*n* = 25) or SY2018 (*n* = 1) (Table 1) and three were not assigned with PBT.

**TABLE 2:** Parentage‐based tagging assignments for the Methow River basin carcass surveys from 2017 to 2020.

PBT assigned hatchery	SY	2017	2018	2019	2020
LNFH	2013	1			
WTP	2014	59			
WTP	2015		31		
LNFH	2016			23	
WTP	2016			24	
WTP	2017				25
WTP	2018				1

*Note*: The PBT‐assigned hatcheries include Leavenworth NFH (LNFH) and Winthrop NFH (WTP) from the various spawn years (SY).

## 4 DISCUSSION

Here we present the first Coho salmon PBT baseline for hatcheries contributing to reintroduction of this extirpated species in the interior CRB. The baseline contains over 32,000 samples from nine hatcheries over a period of 9 years. In addition to identifying the origin and total age of returning offspring, the PBT baseline can also be used to track levels of genetic diversity and relatedness among spawn years, estimate the number of effective breeders, and measure the degree of genetic differentiation among hatcheries and broodlines. The PBT baseline also enables several applications such as evaluating the contribution of hatchery fish to natural spawning in the Methow River basin and mixed stock analyses at Priest Rapids Dam.

### 4.1 Coho Salmon population structure and diversity

The re‐introduction of Coho salmon to the interior CRB has generally been considered a success as the number of adults returning to upriver sites continues to increase and recreational and subsistence fisheries have been opened on many rivers upstream of Bonneville Dam. Now that the first goal of restoring Coho salmon to some of its historic habitat has been achieved, program managers are implementing secondary phases of management, which aim to establish locally adapted stocks of Coho salmon and, ultimately, only use hatchery rearing as a form of supplementation to the natural stock to re‐establish local populations with relatively high fitness. Hatchery‐reared Coho salmon can have lower reproductive success compared with their natural‐origin counterparts, especially for males (Neff et al., 2015; Thériault et al., 2011) and rearing in a non‐natural environment can decrease fitness when allowed to spawn naturally (Frankham, 2005). However, the aim to utilize naturalized Coho salmon from these reintroduction programs may lead to improvements in productivity as has been demonstrated for other salmonid supplementation programs following local extirpation (e.g., Nuetzel et al., 2023). Further, fitness for Coho salmon can differ across hatchery programs with cases where hatchery rearing has not led to significantly lower reproductive success than natural‐origin fish (reviewed in Koch & Narum, 2021). While inbreeding and reductions in fitness are more likely to be observed in captive‐bred populations (Frankham, 2005), only minor deviations in observed heterozygosity were noted in this study (average difference between *H*_*O*_ and *H*_*E*_ of 0.001). There were 18 collections that had positive *F*_IS_ values (95% CI that did not overlap with zero) that may have indicated instances of reduced heterozygosity of approximately 1%. In one case (WTP12), heterozygous excess was significant which could occur in hatchery collections for multiple reasons (small effective population size, relatedness and nonrandom sampling) (Wang, 2018; Waples, 2015). Since *N*_*b*_ estimates for WTP12 were not particularly low and there was no evidence of inbreeding in the subsequent generation in WTP15, this result of low *F*_IS_ was likely due to nonrandom sampling of individuals in the WTP12 spawn year. Nonrandom inclusion of heterozygotes may have occurred in this collection since samples in that year that had higher levels of failed genotypes than any other spawn year. Thus, while reintroductions are generally derived from a sub‐set of a much larger population which can lead to bottleneck‐type effects including genetic drift, loss of heterozygosity and inbreeding effects (Lamothe et al., 2019), those effects were generally not prevalent in these Coho salmon programs.

As expected for reintroduction programs, the genetic structure of reintroduced populations is highly dependent on the source populations (Latch & Rhodes Jr, 2005). With reintroductions of Coho salmon starting in the mid‐1990s, there have been at least eight generations of rearing and returns to sites above Bonneville Dam in the Columbia River basin. Without more complete data from the source populations, it is unknown how much differentiation has occurred since the reintroduction efforts occurred in recent decades. Stock transfers among hatcheries will also affect genetic structure by decreasing the amount of differentiation among geographic locations by increasing gene flow among hatchery programs. The observed genetic structure for the Coho salmon hatcheries was more pronounced among broodlines than among hatcheries, largely due to distinct 3‐year‐old age classes with limited overlapping spawners across ages. This pattern may have been propagated in the lower river source populations as it has been observed before for Coho salmon in the mid‐Columbia River (Campbell et al., 2017) and in hatcheries from Washington state (Smith et al., 2015; Van Doornik et al., 2002) but is not typically observed in natural populations of extant Coho salmon across their range that have more variable age classes (i.e., Beacham et al., 2001; Rougemont et al., 2023; Van Doornik et al., 2007; Xuerub et al., 2022). This study demonstrated that the different broodlines can have different *N*_*b*_ values within a hatchery which may negatively impact those broodlines with smaller values of *N*_*b*_. A decline in *N*_*b*_ is often linked to decreased population size, which can lead to loss of genetic diversity and increased inbreeding depression (Luikart et al., 2021). By perpetuating discrete broodlines in Coho salmon hatchery populations due to limited overlapping age classes included in broodstock (i.e., nearly all three‐year‐old spawners), overall genetic diversity and effective population size can be negatively impacted (Bohling & Von Bargen, 2020; Smith et al., 2015). By retaining distinct broodlines, the increased risk that the complete loss of one broodline could result in the loss of one‐third of the population genetic variability (Smith et al., 2015). In natural populations of Coho salmon, it has been estimated that jack males can contribute 35% of the spawning effort, whereas in a hatchery population jacks contributed only 2% (Van Doornik et al., 2002). In addition, not including jacks in hatchery broodstocks can actually limit the adult female size over time due to negative intersexual heredity (i.e., differences in the patterns of heredity among sexes) from selection against jacks, thereby limiting female reproductive output (Gamble & Calsbeek, 2023). Purposefully allowing more jacks into a hatchery broodstock would increase gene flow among broodlines and would be expected to improve the long‐term diversity and sustainability of reintroduced Coho salmon. For example, inclusion of up to 20% jacks in broodstock has eliminated the restricted gene flow among broodlines in the Quilcene NFH in Washington state in only three generations (Bohling & Von Bargen, 2020). Maintaining the level of jack spawning that is observed in natural systems can help safeguard hatchery stocks against the formation of broodlines and eliminate the potential genetic risks associated with age‐class differentiation. However, some reintroduced programs in this study had very low numbers of returning jack males. For example, of all the adult Coho that have returned to Three Mile Dam from 1989 to 2020, an average of only 11% were jacks (median value of 6%). Alternative breeding tactics (e.g., cryopreserved milt) may be necessary to reestablish gene flow among broodlines and jack males in these programs.

### 4.2 Applications of the PBT baseline

The goal of the mid‐Columbia River Coho salmon re‐introduction program facilitated by the YN is to develop a locally adapted stock that does not rely on hatchery propagation to maintain the population. In some locations such as the Wenatchee River, the YN is replacing the need for CWTs by tracking all adults to their respective progeny with PBT and using the parentage assignments to estimate smolt‐to‐adult return ratios as a cost‐effective alternative to CWT. Additionally, the PBT baseline can aid in developing an estimate of run reconstruction to the Upper Columbia River by routine genotyping of adults that pass Priest Rapids Dam. Carcass surveys, in general, can be used in conjunction with PIT tag detections to help estimate the distribution and range of natural‐origin spawners and the size and age composition of fish on the spawning ground; however, these surveys can underestimate the number of hatchery fish on the spawning ground (Murdoch et al., 2010). Inaccurate estimates of origin due to unmarked fish or low tagging rates can lead to overestimating the number of natural‐origin fish that are reproducing outside of the hatchery environment. The use of PBT for carcass samples can detect and verify both the origin and age of fish on the spawning grounds due to high tag rates and is helping to assist YN in determining the distribution and range of Coho salmon in the mid‐Columbia River basin.

### 4.3 Future applications of the PBT baseline

Future re‐introduction efforts of Coho salmon in the Columbia River are expected to greatly benefit from ongoing PBT baselines. For example, in the Lostine River, a tributary to the Grande Ronde River located in northeast Oregon, the NPT and the Oregon Department of Fish and Wildlife have been working to re‐establish an extirpated population of Coho salmon. They began releases of Coho salmon in 2017 of Tanner Creek stock to the Lostine River that were collected at Bonneville Dam and transferred to Cascade Hatchery for rearing. There has been volitional return of fish to the Lostine River and documented natural spawning through redd counts in the Lostine, Lower Wallowa and Grande Ronde rivers, but future parentage analyses could examine if certain stocks have higher reproductive success than others in this new environment. Another key question that would aid with future funding for this program is to understand the level of dispersal to other watersheds for Coho salmon released in the Lostine River. If all fish released in the Lostine River are PBT tagged, a PBT baseline for this population can be established to track the movement of these fish to other watersheds or their catch in mixed‐stock fisheries.

Another potential application for this Coho salmon PBT baseline would be to detect fish that disperse and attempt to colonize non‐natal areas. At Warm Springs NFH along the Warm Springs River (Deschutes River basin), a location where Coho salmon were extirpated, Coho salmon have been returning in generally increasing numbers in recent years. The hatchery does not have a history of spawning or releasing Coho salmon. While natural productivity has now occurred for several generations, the adults that initially established this population may have been of hatchery origin. If so, PBT analysis could be used to identify the origin of those Coho salmon that dispersed to the Warm Springs River in the early 2010s. This is with the caveat, however, that to ensure accurate assignment and origin inference, all hatchery programs releasing Coho salmon in the interior CRB would need to sample and genotype broodstock to detect dispersing fish.

Since PBT can be used to identify offspring at any life stage, there are also applications for analyzing juvenile Coho salmon. For example, the YN Fisheries program in the Mid‐Columbia River are interested in classifying hatchery and natural‐origin Coho salmon juveniles during their outmigration. Analyses based on PBT would develop better estimates of egg‐to‐emigrant survival and help monitor and/or manage the proportion of natural origin spawners in this region where complete broodstock sampling has occurred for Coho salmon hatchery programs for multiple generations. Coho salmon juvenile fin clips have been collected from various screw traps in the Methow and Wenatchee River basins and enabling the use of the existing PBT baseline to classify natural and hatchery‐origin samples.

Finally, the application of the Coho salmon PBT baseline can be used to identify individual fish in mixed stock fisheries, either harvested in‐river or in the ocean. For an in‐river harvest example, it is of interest to YN Fisheries to determine the contribution of Mid‐Columbia River Coho salmon that are harvested in the zone 6 fishery along the Columbia River that contains a mix of upriver stock. This fishing zone, stretching from Bonneville to McNary Dam on the Columbia River, is exclusively limited to fisheries of the treaty tribes for Coho salmon. In a similar manner, the origin of Coho salmon could be identified in the ocean harvest. Additional stocks other than interior CRB hatcheries would be encountered so a combination of PBT and traditional genetic stock identification (GSI) tools would be needed (e.g., Beacham, Wallace, Jonsen, McIntosh, Candy, Willis, Lynch, Moore, et al., 2019) with further representation of all contributing stocks. Thus, there would need to be greater coordination from lower Columbia River and coastal hatcheries and other state and federal institutions to standardize PBT and GSI baselines for a common SNP panel that could be used across a broader geographic area (i.e., Rougemont et al., 2023; Van Doornik et al., 2007; Xuerub et al., 2022).

## 5 CONCLUSIONS

Parentage‐based tagging is widely used in the Columbia River basin for Chinook salmon (*O tshawytscha*) and steelhead (*O. mykiss*), but its utility for Coho salmon has been limited. Besides the ability to track broodstock composition, PBT baseline data can be used to characterize the level of genetic diversity, population differentiation and number of breeding individuals, which are all important metrics to track over time. The broader goal would be the consistent collection of broodstock from all Coho salmon hatcheries in the basin, but reintroduction efforts in the interior Columbia River will be aided by continued monitoring of the YN, CTUIR and NPT's programs.

## CONFLICT OF INTEREST STATEMENT

The authors have no conflicts of interest to declare.

## Supporting information


Figure S1.

Table S1.

Table S2.

## Data Availability

Data for this study (samples, their metadata and genotypes) are available on FishGen.net. To download data, users must request an account (https://www.fishgen.net/WebPages/User/UserRegistration.aspx).
