# Supplementary material for: Utility of parentage‐based tagging for monitoring Coho salmon (Oncorhynchus kisutch) in the interior Columbia River basin
Source: Evol Appl. 2023 Dec 8;17(2):e13607. doi: 10.1111/eva.13607 (PMC10853591; doi:10.1111/eva.13607)
Supplement: Supplementary file 1 — Figure S1. [file EVA-17-e13607-s003.docx]

Utility of parentage-based tagging for monitoring Coho salmon (*Oncorhynchus kisutch*) in the interior Columbia River basin

Horn et al. Supplemental Figures


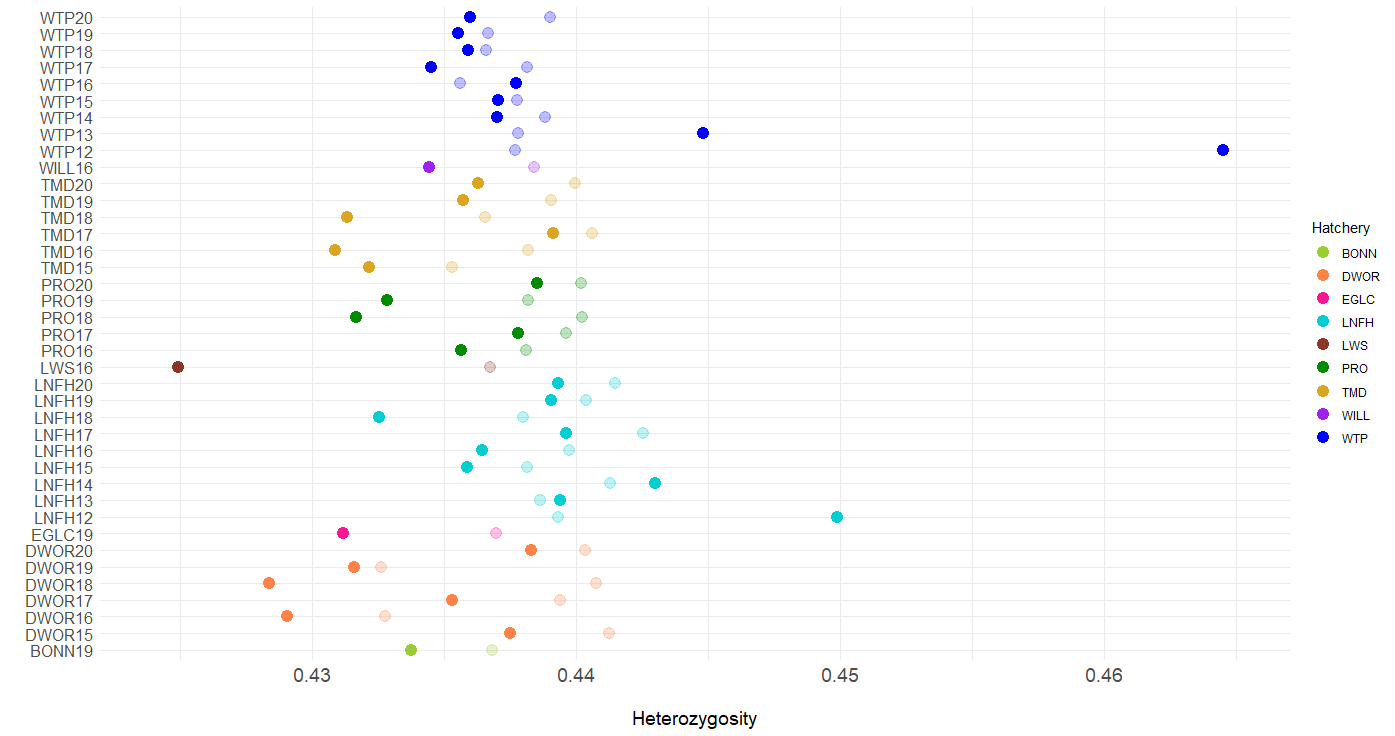


Supplemental Figure 1. The observed (solid-colored circle) and expected (light-colored circle) heterozygosity for each hatchery and brood year. A list of hatchery abbreviations can be found in Table 1.


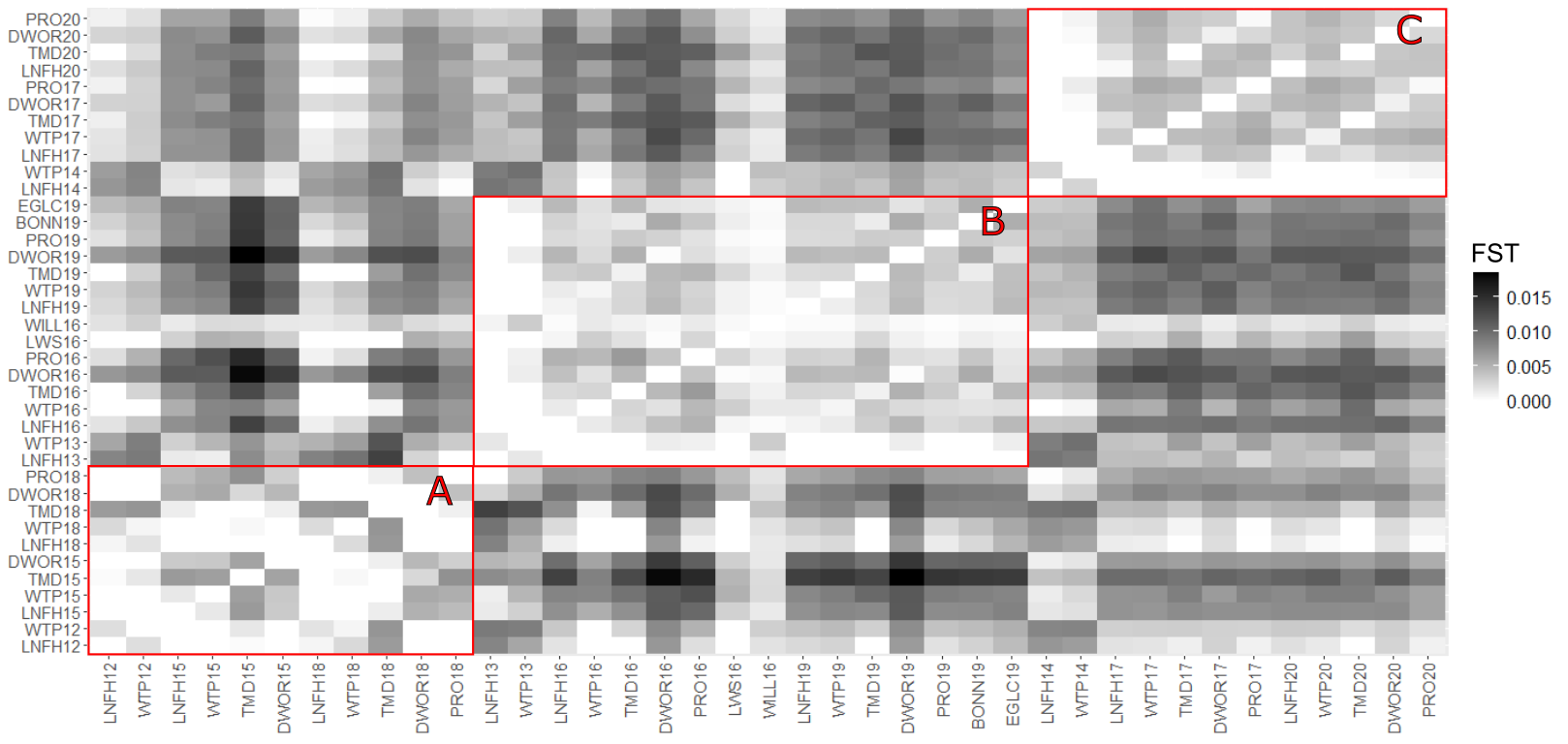


Supplemental Figure 2. A heat map of the pairwise FST values among all Coho salmon hatcheries and brood years. The red boxes and letters represent the “A” broodline (spawn years 2012, 2015, 2018), the “B” broodline (2013, 2016, 2019), and the “C” broodline (spawn years 2014, 2017, 2020). For a list of hatchery abbreviations, see Table 1.


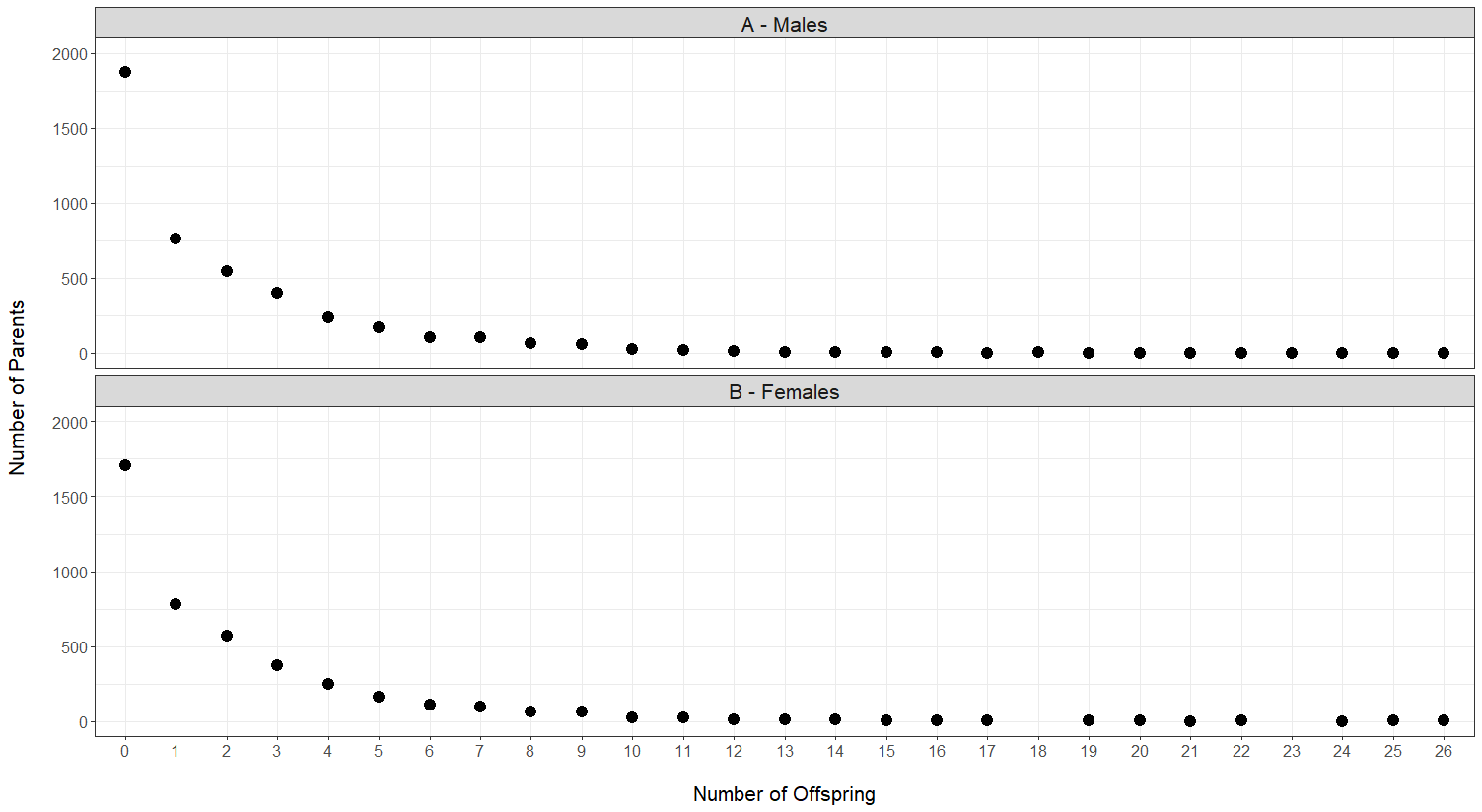


Supplemental Figure 3. The number of offspring produced by male (A) and female (B) Coho salmon from 2012-2018.
